
JOURNAL INFORMATION
==============================
NLM Title Abbreviation: HPB Surg
NLM Journal ID: 9002972

HPB Surgery

ISSN: 0894-8569
EISSN: 1607-8462
Publisher: Wiley

ARTICLE INFORMATION
==============================
PMCID: PMC2423499
PMID: 3153771
DOI: 10.1155/1988/81529
Article ID: S0894856988815297
Article version: 1
Subjects: Research Article

Editorial

Bengmark Stig
Print publication date: 1988
Volume: 1
Issue: 1
First page: 1
Last page: 2
Copyright: Copyright © 1988 Hindawi Publishing Corporation.
Copyright year: 1988
Copyright holder: Hindawi Publishing Corporation
License: This is an open access article distributed under the Creative Commons Attribution License, which permits unrestricted use, distribution, and reproduction in any medium, provided the original work is properly cited.
License URL: http://creativecommons.org/licenses/by/

==============================
*HPB Surgery* 1988, Vol. 1, pp. 1–2
Reprints available directly from the publisher
Photocopying permitted by license only

© 1988 Harwood Academic Publishers GmbH
Printed in Great Britain

### EDITORIAL

Surgical diseases of the liver, pancreas and biliary tract are very frequent and some are on the increase. The liver is only second to the lymph nodes, the area in the body which is the most frequent site of tumor growth. Manifestations occur in the liver in at least 1/4 of all patients with cancer. Approximately 50% of patients with cancer of the digestive tract, sooner or later contract hepatic lesions aswell. In the West approximately 90% of the tumors are of secondary nature whilst in some developing countries 90% of the tumors are of primary nature, the sum, however, is the same. Although gall-stone disease in some countries is decreasing, it is still one of the most frequent surgical diseases in the Western world. There was a time when approximately 10% of surgical beds were occupied with patients with biliary diseases in some Western countries. The incidence of pancreatic cancer and liver cirrhosis/portal hypertension is also increasing.

The diagnostic arsenal has changed tremendously during recent years. The availability of ultrasonography, CT and NMR makes it easier to diagnose malignant diseases in these organs at a time when they are still treatable. The ultrasound endoscopy will add further improvement. Today, no more than 20% at the most, of diagnosed tumors in the liver and 10% in the pancreas can be surgically resected. This will supposedly increase during the years to come. In addition, ultrasonography especially is becoming easily available. Small portable and, compared with other tools, rather inexpensive machines for ultrasonography are available. This makes it possible to use this technique in small and periferal units as well as in different parts of the larger hospital; emergency room, operation department(s) and the wards.

Due to the availability of new tools to be used during operations, such as ultrasonography, ultrasound dissectors, jet dissectors, solid organ staplers etc., operations have become more complex though often easier. In addition, success in this rather extensive surgery depends much upon the use of good knowledge in pathophysiology and good treatment in intensive care. The availability of sound knowledge about the interior anatomy of the liver, the functions of the sphincter of Oddi, the vascularization of the organs, the spreading of tumors through different groups of lymph nodes has made it possible to select suitable operations for each individual.

Not long ago, surgery of the liver, pancreas and biliary system was rather stereotype. This has changed dramatically. There are today alternative techniques; non-invasive operations, percutaneous or endoscopical, chemical techniques like dissolution of gall-stones etc. In a few areas the cooperation between different specialties has developed to a great extent such as the area of the pancreatic and biliary system. Sound cooperation between physicians, endoscopists, radiologists, pathophysiologists, clinical physiologists, microbiologists, pathologists and cytologists is of extreme importance for the success of this type of surgery. More and more centres for HPB surgery are now being established around the world.

The rapid development within the field has made it necessary for surgeons and physicians to have forums for exchange of knowledge and discussions. In this pluralistic medical world there is a need for many different attempts to improve communications. Until now no journal with a special interest in the surgery of the liver, pancreas and biliary system has existed. We are very thankful to Harwood Academic Publishers GmbH for their initiative to fill this gap by establishing the present journal. It is the hope of the Editors and the Editorial Board that the journal will be successful. Success, however, in the end, depends on the contributors.

Stig Bengmark
